# Animal Model Selection and Deep Phenotyping in Mitochondrial Genetic Diseases: Connecting Human Variants, Multisystem Pathology, and Preclinical Evidence

**DOI:** 10.3390/genes17101165

**Published:** 2026-09-22

**Authors:** Nobuya Sasaki, Masaki Watanabe

**Affiliations:** Laboratory of Laboratory Animal Science and Medicine, School of Veterinary Medicine, Kitasato University, Towada 034-8628, Aomori, Japan; watanabe.masaki@kitasato-u.ac.jp

**Keywords:** mitochondrial disease, animal model, deep phenotyping, mouse model, mtDNA editing, heteroplasmy, preclinical research, reproducibility, 3Rs, translational research

## Abstract

Mitochondrial genetic diseases arise from variants in mitochondrial DNA (mtDNA) or nuclear genes required for mitochondrial function. Although many involve several organs, the predominant clinical manifestations may be confined to particular organs. Experimental models are used to investigate variant pathogenicity, organ vulnerability, disease progression, therapeutic response, and safety. We discuss how to select animal models and other experimental systems for specific research questions. We focus on genetically engineered mice (constitutive knockout, knock-in/humanized, conditional/tissue-specific, inducible, and heteroplasmic mtDNA models) and consider when yeast, worms, flies, zebrafish, rats, large animals, companion animals, and human in vitro models can complement or replace particular animal experiments. We define deep phenotyping by five minimum characteristics: longitudinal assessment across defined disease stages; prespecified relationships among molecular, mitochondrial, organ, and whole-animal measurements; integration of repeated and terminal endpoints; alignment with patient-relevant disease stages; and separation of primary from exploratory outcomes. Model selection depends on the experimental objective and the evidence needed to address it. We also propose a multidomain framework that distinguishes target engagement, molecular correction, downstream pharmacodynamic responses, organ function, and whole-animal or patient-relevant benefit without assuming that effective treatments must improve every domain sequentially. We also discuss reporting practices, the 3Rs (Replacement, Reduction, and Refinement), and the implications of mtDNA editing, iPSC-derived systems, organoids, and automated phenotyping for study design.

## 1. Introduction

Mitochondrial genetic diseases are diverse inherited disorders caused by abnormalities in mitochondrial DNA (mtDNA) or in nuclear genes that support mitochondrial function [1]. Many nuclear genes encode proteins required for mitochondrial biology. Patient variants may disturb oxidative phosphorylation (OXPHOS), mitochondrial protein translation, mitochondrial dynamics, quality control, metabolite transport, or mtDNA maintenance [1,2]. Because mitochondria support ATP production, reactive oxygen species homeostasis, calcium signaling, apoptosis, and intermediary metabolism, mitochondrial dysfunction can disrupt several organ systems and lead to diverse clinical manifestations [1,2].

Although mitochondrial diseases frequently involve multiple organs, tissue vulnerability and clinical presentation can be highly organ-selective [1]. Tissues with high energy demand, including the brain, skeletal muscle, heart, liver, kidney, retina, auditory system, and endocrine organs, are commonly affected [1]. Clinical presentations range from fatal neonatal metabolic failure to slowly progressive adult-onset neurodegenerative syndromes [1]. Even among patients who carry variants in the same disease-related gene, severity, age at onset, and affected organs can vary widely because of heteroplasmy, tissue-specific energy demands, nuclear genetic background, environmental factors, and stochastic biological variation [1,3]. This variability is a major challenge for diagnosis, prognosis, and therapeutic development.

Animal models are central to mitochondrial disease research [2,4]. Genetically engineered mice allow precise manipulation of disease-related genes and make it possible to analyze physiology, pathology, metabolism, and behavior from development through adulthood. This makes them a major experimental platform for mitochondrial disease research and preclinical evaluation [2,4,5]. Knockout, knock-in, conditional, inducible, and tissue-specific mouse models have shaped the current understanding of neurodegeneration, cardiomyopathy, myopathy, liver dysfunction, sensory phenotypes, and metabolic abnormalities [2,4,6,7,8]. CRISPR/Cas9 genome editing has further accelerated the study of nuclear-encoded mitochondrial genes and the experimental testing of patient variants [9].

Yet reproducing a candidate causal variant does not automatically yield a clinically informative model [2,4,10]. Genetically engineered animals may show molecular abnormalities without the symptoms or functional deficits that matter to patients [2,4,6,7,8,10,11,12,13]. Conversely, animals with different genetic lesions can converge on similar pathology, indicating that mitochondrial dysfunction is shaped by shared biological pathways as well as gene-specific mechanisms [2,4,6,7,10,11,12,13]. Useful models therefore need integrated evaluation across the molecular, cellular, organ, and whole-animal levels, not only confirmation of genotype or a single molecular abnormality.

Advances in phenotyping have changed how animal models are evaluated [1,8]. Earlier studies often relied on endpoints such as survival, body weight, histopathology, and enzyme activity [1,8]. These measures remain essential, but they do not always capture the dynamic, multisystem nature of mitochondrial disease [1]. Mitochondrial dysfunction can affect cellular metabolism, neural function, cardiovascular physiology, immune responses, and whole-animal behavior at the same time [1,8]. Following these changes over time can help relate early molecular abnormalities to tissue injury and subsequent loss of function.

Deep phenotyping can also improve reproducibility. Reproducibility has become a major concern in studies using complex animal models [14,15]. Genetic background, microbiota, environment, husbandry, experimental procedures, and analytical methods can all influence phenotype [14,15,16]. Well-documented phenotyping protocols reduce unexplained variation, make inter-facility comparisons easier, and increase confidence in the results [14,15,16,17]. International efforts to standardize phenotyping and data sharing are therefore central to modern animal research [5,17,18].

This review addresses four practical decisions in mitochondrial disease research: selecting a model, choosing appropriate endpoints, interpreting agreement or disagreement between models, and assessing the evidence for a therapeutic effect. We bring genetic design and longitudinal phenotyping together to help investigators plan studies and judge what their results can support.

Patient data, biomarkers, induced pluripotent stem cell (iPSC)-derived systems, and organoids are discussed where they inform model selection or endpoint design. Clinical management was outside the scope of this review.

This article is a narrative and methodological review rather than a systematic review. PubMed and Web of Science were searched through 31 August 2026 using combinations of the terms “mitochondrial disease”, “animal model”, “mouse”, “rat”, “pig”, “yeast”, “zebrafish”, “organoid”, “iPSC”, “deep phenotyping”, “heteroplasmy”, “mtDNA editing”, “gene therapy”, “preclinical”, and the names of representative disease genes discussed in the text. English-language original studies and major reviews were considered. We prioritized studies that illustrated allele specificity, tissue or temporal restriction, heteroplasmy, longitudinal phenotyping, cross-species discordance, patient-endpoint translation, therapeutic response, or safety. Clinical-management studies without a model-design or endpoint-design component, studies lacking sufficient methodological information for the point being discussed, and redundant examples addressing the same design principle were not emphasized. Citation tracking from major reviews was used to identify additional primary studies. Studies were selected qualitatively for their relevance to these methodological questions. The search was not exhaustive and is subject to selection bias.

### Terminology Used in This Review

A model system is a broad experimental framework that includes cultured cells, organoids, model organisms, and animal models. A model organism is a living organism used to study genes or disease biology, whereas an animal model is an animal-based system used to investigate disease mechanisms or therapeutic responses. Mammalian models include mice, rats, pigs, dogs, and cats. Deep phenotyping is a hypothesis-driven strategy linking molecular changes, mitochondrial function, tissue pathology, organ function, and whole-animal outcomes over time; it does not simply mean collecting more measurements. Reproducibility refers to confirmation of the main conclusion in an independent cohort, experimental batch, facility, or complementary model, as appropriate. As summarized in Figure 1, model selection should begin with the claim to be tested because variant pathogenicity, organ vulnerability, disease progression, therapeutic efficacy, and safety require different designs, controls, and primary endpoints. Complementary experimental systems are summarized in Figure 2.

## 2. Genetic Construction and Classification of Animal Models

Animal models have been central to understanding mitochondrial disease pathophysiology and evaluating new treatment strategies [2,4,10,11,12,13]. Early models and non-targeted experimental systems included strains generated by spontaneous mutation, gene-trap methods, or ENU mutagenesis. These models reproduced some aspects of mitochondrial dysfunction and contributed to mitochondrial biology, but their relationship to specific causal genes or patient variants was sometimes unclear. This limited their value for direct testing of the pathogenicity or treatment response of a particular patient variant [2,4,11,12,13].

Gene targeting in embryonic stem cells enabled knockout mice lacking a defined gene and knock-in mice carrying a specific mutation, allowing the effects of defined genetic changes to be tested [4]. More recently, CRISPR/Cas9 has accelerated model generation and functional validation of nuclear-encoded mitochondrial genes [9]. Together, these technologies have shifted animal models from gene-discovery tools toward experimental validation of variants found by patient genetic analysis [4,9,10].

The value of a genetically engineered model depends on how its phenotype relates to the intended experiment [2,4,10,12,13]. Tissue specificity, disease progression, and interactions between nuclear and mitochondrial genomes can all affect that relationship [1,3]. These features should be considered when selecting an allele and deciding where and when to express it [2,4,10,12,13].

Genetically engineered mitochondrial disease models are better viewed along several design axes than as mutually exclusive categories. One axis is allele design: knockout, knock-in, humanized allele, or mtDNA variant. A second axis is spatial control: systemic, tissue-specific, or cell-type-specific manipulation. A third axis is temporal control: constitutive or inducible perturbation, such as CreERT2 or Tet-On/Tet-Off. A fourth axis is mtDNA status: homoplasmy, heteroplasmy, or manipulation of mutant mtDNA load. In practice, a single model may carry several attributes; for example, a patient-variant knock-in may also be tissue-specific or inducible. Table 1 summarizes how these designs can be used according to research purpose [2,4,9,12,13].

### 2.1. Constitutive Knockout Models

Constitutive knockout mice are well-suited for examining the in vivo roles of nuclear genes involved in mitochondrial biology [2,4,6,7]. Because gene function is lost throughout the organism from early development, these models can reveal how strongly a gene is required for embryogenesis, tissue maintenance, and systemic metabolism [2,4,6,7].

Deleting genes involved in OXPHOS, mtDNA replication, mitochondrial protein translation, or mitochondrial quality control often causes embryonic lethality [2,4,6,7,19]. Such models establish a developmental requirement for the gene, but their early lethality prevents the investigation of postnatal progression or adult organ dysfunction.

Viable constitutive knockout models have also been informative [2,4,20]. For example, Surf1-deficient mice show COX deficiency but do not fully reproduce the spontaneous neurodegeneration seen in human Leigh syndrome [20]. This example shows that deletion of a disease gene does not necessarily reproduce the corresponding human phenotype. In viable null models, compensation and species differences can strongly shape the observed phenotype.

These strengths come with limitations, including developmental compensation, activation of alternative metabolic pathways, embryonic lethality, and mismatch between complete loss of function and the partial loss-of-function or missense variants common in patients [2,4]. For this reason, constitutive knockouts are now often paired with genetic approaches that control when or where gene disruption occurs [4,9].

### 2.2. Knock-In and Humanized Models

Knock-in models place patient variants into the genome of mice or other organisms [4,9,10]. Unlike knockout models, which abolish gene function, knock-in models usually express the mutant protein under near-endogenous regulation. They are therefore useful for examining the molecular consequences of patient variants [4,9,10].

Patient-specific knock-in models are valuable for studying genotype–phenotype relationships, variant pathogenicity, incomplete penetrance, and variability in severity [4,9,10]. Many mitochondrial diseases are caused not by complete loss of a gene but by missense variants that partially reduce protein function. Knock-in strategies are therefore often better suited than conventional knockout models for studying hypomorphic alleles [1,4,10].

Deep phenotyping increases the value of patient-variant models [8,10]. Molecular assays can detect subtle metabolic changes before symptoms become obvious. Longitudinal physiological assessment can identify progressive decline that a single time point would miss [8,21,22]. Behavioral and sensory testing may also reveal early functional changes before overt neurodegeneration or cell loss, helping to define therapeutic windows [8,21,23].

Knock-in models require checks that the introduced allele behaves as expected. Expression, splicing, protein abundance, localization, and biochemical effects should be confirmed [9]. A mild phenotype may reflect genuine residual function, but it may also reflect species-specific compensation or failure of the allele to reproduce the molecular effect of the human variant [4,9,10]. Unexpectedly severe phenotypes may reflect a susceptible genetic background, founder-line effects, or experimental conditions [9,14,15]. Littermate controls, isogenic controls when feasible, and replication in an independent line or cohort should be considered.

### 2.3. Conditional and Tissue-Specific Models

Constitutive knockout models have clarified many functions of mitochondrial genes, but many mitochondrial proteins are essential for embryogenesis [2,4,6,7]. Whole-body deletion often causes embryonic or perinatal lethality, preventing the analysis of postnatal disease progression or organ-specific pathology [2,4,6,7]. Conditional knockout models were developed to overcome this limitation by controlling gene inactivation in space, time, or both [2,4,21,24,25].

The Cre-loxP system is widely used to generate conditional mitochondrial disease models [2,4,21,24,25]. By crossing mice carrying a floxed allele with tissue-specific or inducible Cre driver lines, the target gene can be inactivated in a selected organ, cell type, or developmental period [2,4,21,24,25]. This permits the study of mitochondrial dysfunction in mature tissues while avoiding developmental defects that could mask disease mechanisms [2,4,21,24,25].

Conditional knockout studies show that the site of mitochondrial dysfunction strongly shapes phenotype [21,22,24,25]. Nervous system deletion of Ndufs4 results in prominent progressive neurodegeneration and breathing abnormalities [23,26,27], whereas cardiac or skeletal muscle deletion of Tfam and skeletal muscle or liver deletion of Cox10 cause cardiomyopathy, myopathy, or hepatocellular pathology [21,24,25,28,29]. L7-Cre-mediated conditional deletion of Mfn2 in cerebellar Purkinje cells showed that mitochondrial fusion is required for the dendritic development, maintenance, and survival of mature Purkinje cells [22]. These examples demonstrate that tissue-specific energy demand, developmental stage, and compensatory capacity can determine the dominant pathology [6,7,21,22,24,25].

One strength of conditional models is that they allow for the temporal analysis of disease progression [21,24,25]. By choosing the Cre driver and target cell type, investigators can partially separate developmental effects from adult degenerative processes. Longitudinal analysis can help place molecular changes, structural pathology, physiological dysfunction, and symptoms in order, thereby informing the timing of therapeutic intervention [21,24,25].

Conditional models still require caution. Cre recombination efficiency and specificity differ among driver lines, and deletion may be incomplete or mosaic [2,4]. Cre or CreERT2 activity itself can cause DNA damage, reduced proliferation, or organ-specific toxicity independently of the target gene deletion [30,31]. Appropriate Cre-negative, Cre-positive, and inducer-treated controls are therefore required, along with confirmation of recombination efficiency and assessment of Cre-related toxicity [17,30,31].

Moreover, conditional deletion is still usually a complete loss-of-function approach, whereas many human mitochondrial diseases are caused by hypomorphic or missense variants with residual activity [1,4]. Conditional knockout results should therefore be interpreted with knock-in or humanized models when possible.

Tissue-restricted models test whether mitochondrial dysfunction in a particular cell population or organ is sufficient to generate a given pathology. The Cre driver, onset of Cre activity, mosaicism, and off-target expression should be reported and experimentally verified [4,17]. Comparing the same floxed allele across neuronal, cardiac, skeletal muscle, and hepatic Cre drivers is especially useful because differences can then be attributed more confidently to tissue environment rather than allele construction [6,7,8,21,24,25].

### 2.4. Inducible Models

Tissue-specific models determine where a mitochondrial gene is perturbed, whereas inducible models determine when the perturbation begins [2,4]. This temporal control is important because many mitochondrial diseases involve postnatal or age-dependent onset and progression in addition to congenital abnormalities [1,4]. Inducible models can separate the developmental functions of mitochondrial genes from their physiological roles in mature tissues [2,4].

Common inducible systems include tamoxifen-inducible CreERT2 and tetracycline-regulated Tet-On/Tet-Off systems [2,4]. These approaches can activate or inactivate a target gene at a selected developmental stage or experimental time point, allowing closer modeling of postnatal disease onset [2,4].

Inducible models are especially useful for studying the temporal sequence of pathogenesis [2,4]. Time-course analysis after induction can test whether molecular abnormalities precede structural pathology or physiological dysfunction. Behavioral changes and clinical signs can then be placed on the same disease timeline [2,4].

Preclinical studies using longitudinal functional and survival outcomes show that therapeutic effects can depend on disease stage and treatment timing [32,33,34,35]. Treatment before irreversible tissue remodeling may preserve organ function, whereas later intervention may improve molecular biomarkers without fully restoring physiology [10]. Inducible or slowly progressive models are therefore valuable for defining therapeutic windows and optimizing treatment strategy.

Inducible systems need careful design. Recombination efficiency can vary across tissues, induction protocols can introduce experimental variation, and the inducer itself can complicate phenotype interpretation [2,4,30,31]. In tamoxifen-inducible CreERT2 models, dose, route, timing, animal age, and tissue distribution can produce toxicity or hematological effects independent of the target gene [31]. Accurate interpretation requires inducer-only controls, Cre-only controls, Cre-positive/flox-negative controls, and verification of recombination efficiency [17,30,31].

### 2.5. mtDNA Mutation and Heteroplasmy Models

Most mitochondrial disease models target nuclear genes, but the mammalian mitochondrial genome encodes 13 polypeptides that are core subunits of the OXPHOS system, in addition to 22 tRNAs and 2 rRNAs [1,11,12]. Pathogenic mtDNA variants are also important causes of inherited mitochondrial disease [1,11,12]. Modeling mtDNA mutations is therefore a major goal of mitochondrial disease research [11,12].

mtDNA creates experimental challenges that differ from those of the nuclear genome [3,11,12]. Each cell contains many mtDNA copies, normal and mutant mtDNA can coexist in the same cell or tissue, and this state is called heteroplasmy. mtDNA is also transmitted mainly through the maternal lineage [3,11,12]. Precise manipulation of mtDNA is therefore more difficult than conventional nuclear genome editing [12,36,37,38].

Mitochondrial replacement models carrying disease-related mtDNA mutations, often called mito-mice, have clarified the relationship between mutant mtDNA load and disease severity [36,39,40]. These studies demonstrated threshold effects, in which clinical phenotype emerges only when mutant mtDNA exceeds tissue-specific levels [12,36,39,40]. Deep phenotyping has further shown that heteroplasmy can differ among organs and among cell types within a tissue, contributing to clinical heterogeneity [3,12,36,37,38,39,40,41].

Technologies for the direct manipulation of mtDNA have also advanced. Mitochondria-targeted zinc-finger nucleases (mtZFN) and mitochondria-targeted TALENs (mitoTALENs) selectively cut mutant mtDNA and reduce mutant load. DddA-derived cytosine base editors (DdCBEs) and TALE-linked deaminases (TALEDs) introduce base substitutions without cutting mtDNA. These technologies expand the possibilities for generating or modifying mtDNA disease models and controlling heteroplasmy [42,43,44,45,46,47,48,49]. TALED enables A-to-G conversion and broadens the range of mtDNA variants that can be modeled or potentially corrected beyond those accessible to DdCBE [49]. However, the editing window, bystander editing, mtDNA off-target effects, nuclear off-target editing, tissue delivery, and safety must be carefully evaluated for both model generation and therapeutic application [48,49,50].

In heteroplasmy models, sampling strategy is critical [3,12,36,37,38,39,40,41]. The average mutant load in whole tissue can obscure the mutant burden in disease-relevant cell populations because of cell-to-cell segregation, clonal expansion, or loss of severely damaged cells [3,12,36,37,38,39,40,41]. Mutant load should be measured in each relevant tissue, and when possible, in purified cell populations or single muscle fibers. Longitudinal interpretation should consider biased transmission during oogenesis or early embryogenesis, age-related shifts in mutant load, and apparent intervention effects that may reflect altered cellular composition rather than the direct restoration of mitochondrial function [3,12,36,37,38,39,40,41].

## 3. Question-Driven Model Selection and Combination of Model Systems

Figure 1 links the primary research question to allele design, the site and timing of genetic manipulation, endpoints, and controls. Figure 2 shows where other experimental systems can contribute to the same study.

The genetic designs discussed above impose different practical constraints [2,4,10,12,13]. Constitutive knockouts are useful for basic gene-function studies but are often limited by embryonic lethality or developmental compensation [2,4,10,12,13]. Knock-in and humanized models better reproduce patient genotype and are well-suited to translational research, although phenotypes may develop slowly and require long-term longitudinal assessment [2,4,9,10,12,13]. Conditional and tissue-specific models are useful for organ-selective pathology, whereas inducible models allow for the temporal analysis of disease progression and therapeutic intervention [2,4,10,12,13].

The choice also depends on what the experiment must establish [10]. A pronounced knockout phenotype may be useful for dissecting a mechanism, whereas testing a treatment for a particular patient variant may require a knock-in or humanized allele [10]. Biomarker studies need repeated measurements over a suitable disease course. Tissue-specific or inducible models can help resolve the origin and timing of organ injury.

Combining these approaches allows investigators to test findings under different genetic and physiological conditions.

### 3.1. Model Organisms and Large Animals That Complement Mice

No single species or experimental system can assess variant function, multisystem pathology, natural history, pharmacokinetics, and safety with equal precision. The appropriate role of a non-animal or non-mouse system depends on the question. Yeast, human cells, and organoids can replace animal experiments for some questions, including initial variant-function testing, pathway analysis, and selected rescue or screening studies. Whole-animal models remain necessary when the claim concerns integrated physiology, inter-organ interactions, development, pharmacokinetics, long-term natural history, or systemic safety. This question-dependent use is consistent with Replacement: an animal experiment should be used only when a non-animal system cannot provide the evidence required for the prespecified claim [2,17].

*C. elegans* and Drosophila have short generation times, powerful genetic tools, and suitability for large-scale screening. They are useful for studying mitochondrial stress responses, locomotion, neurodegeneration, lifespan, and modifier genes [2,51,52,53]. However, they cannot directly evaluate mammalian cardiopulmonary, renal, or hepatic function, and they cannot by themselves validate patient multisystem pathology. Zebrafish provide transparent embryos, rapid development, in vivo imaging, and relatively large-scale drug screening, making them useful for developmental, neurological, and motor phenotypes associated with mitochondrial dysfunction [2,54]. Differences in ectothermy, gene duplication, gill respiration, and drug exposure routes prevent direct extrapolation to mammalian exposure or organ physiology.

Rats provide practical advantages over mice for repeated blood sampling, surgery, physiological monitoring, pharmacokinetics, and complex behavioral assessment because of their larger body size, although mitochondrial disease alleles and shared genetic resources remain more limited [55]. A recently generated rat carrying an engineered heteroplasmic mtDNA variant developed hearing impairment, illustrating how rat size and auditory phenotyping can support the longitudinal analysis of an mtDNA disease phenotype [56]. Pig models can also reveal disease features that are less apparent in mice. Surf1-null mice show cytochrome c oxidase deficiency but relatively mild spontaneous neurological disease [20], whereas cloned SURF1-knockout pigs developed failure to thrive, muscle weakness, high perinatal mortality, and a markedly shortened lifespan [57]. The contrast shows how strongly species can influence disease severity. Pigs remain costly and require specialized facilities, while naturally occurring diseases in companion animals may add clinically managed natural history and treatment-response data but introduce case heterogeneity, environmental variation, and ethical constraints [58,59].

Experiments can move between these systems as new findings emerge (Figure 2). Patient observations may prompt an animal study, while results in animals may require testing in patient-derived cells. Table 2 summarizes the questions each system can address, together with its practical limitations.

### 3.2. Selection Criteria According to Research Purpose

When evaluating variant pathogenicity, the first requirement is correspondence between the introduced change and the molecular consequence observed in patients [1,4,9,10]. If a variant is expected to retain partial activity, alter protein stability, or exert a dominant effect, a knock-in model is useful [4,9,10]. A null allele can show whether a gene is required for viability or tissue function, but it cannot by itself prove that a specific missense variant causes disease. If the main question is which organ or tissue is vulnerable, conditional models often allow organ-specific effects to be separated from systemic disease [4,21,24,25].

Natural history studies require models with a measurable interval between early functional disturbance and advanced disease [4,10,64]. Models that die before meaningful longitudinal assessment can still be valuable for mechanism, but they are poorly suited to defining a therapeutic window. Inducible alleles can reveal temporal order, whereas slowly progressive knock-in or humanized models can capture mild transitions that severe knockout models miss [4,10,64]. Sampling and assessment should be set by disease stage, not only by chronological age.

Biomarker discovery and intervention studies have additional requirements [10]. Candidate biomarkers should change reproducibly with disease stage and be measurable by procedures that do not substantially alter the phenotype [10]. Therapeutic studies need models in which the target pathway is actually impaired, the drug or therapeutic vector reaches the relevant organ, and the selected functional endpoint can change within the treatment period [10]. A molecular-level positive response in an unsuitable model should not be interpreted as evidence of likely patient benefit. These considerations are summarized in Table 3.

## 4. Case Study: IARS1 as a Cross-System Allelic Series

IARS1 (human; Iars1 in mouse) encodes cytosolic isoleucyl-tRNA synthetase; IARS2 encodes the mitochondrial enzyme [65]. We included IARS1 as a brief comparison because studies of different alleles and species distinguish the partial loss of activity from complete gene deletion and reveal secondary mitochondrial or metabolic abnormalities. Primary mitochondrial disease models, including NDUFS4, POLG, TWNK, TK2, MPV17, OPA1, and heteroplasmic mtDNA models, are discussed in Section 7.

The naturally occurring IARS1 p.Val79Leu allele causes perinatal weakness and low body weight in Japanese Black cattle [63]. The corresponding hypomorphic mouse allele causes growth retardation and hepatic and cellular mitochondrial abnormalities, whereas complete Iars1 deletion causes early post-implantation lethality [66,67]. A different compound-heterozygous mouse model showed pulmonary surfactant accumulation associated with impaired alveolar-macrophage lysosomal and autophagic function [68]. Together with human cases and yeast complementation [69], these findings illustrate how residual activity and cellular context affect the consequences of IARS1 deficiency (Table 4).

## 5. Experimental Factors That Shape Phenotype

Genetic background, heteroplasmy, sex, age, and experimental conditions can modify both the disease phenotype and its response to treatment (Figure 3). Recording these variables is essential for interpreting differences between cohorts or laboratories.

Genetic background is a major modifier of penetrance and severity [14,15]. Differences in nuclear-encoded respiratory chain components, stress responses, metabolism, immunity, and behavior can produce different phenotypes from the same allele. Strain, substrain, breeding strategy, backcross generation, and the use of littermate controls should therefore be reported [14,15,17]. Sex and age should also be incorporated into allocation and analysis. If only one sex is used, the limits of generalizing to the other sex should be stated [14,15,17].

In mtDNA models, heteroplasmy determines disease susceptibility and severity, but heteroplasmy itself can vary by littermate, tissue, cell type, age, and disease stage [3,11,12,36,37,38,39,40,41]. Nuclear background and maternal lineage can also influence the transmission and tissue distribution of mutant mtDNA. Groups should be balanced for baseline heteroplasmy, and conclusions should be based on the mutant load in the affected organ rather than only in readily sampled tissues such as blood. Disease should be analyzed as a continuous relationship between mutant mtDNA load and functional impairment, not simply as mutant-positive or mutant-negative [3,11,12,36,37,38,39,40,41].

Housing temperature, diet, fasting duration, circadian phase, cage density, environmental enrichment, microbiota, and handling can alter energy expenditure and metabolic stress [15,16,70,71]. Anesthesia, restraint, forced exercise, fasting, and invasive sampling can also affect respiration, body temperature, cardiovascular function, substrate metabolism, and stress responses [17,71]. Study design should therefore distinguish disease-related changes from physiological responses to measurement procedures through pilot studies, sham-procedure controls, temperature management, balanced test order, and statistical analysis that reflects hierarchical structure [14,15,17,71]. Multisite replication and controlled biological heterogeneity can provide stronger evidence for generalizability than extreme standardization within a single facility [14,15,16].

## 6. Longitudinal Deep Phenotyping Across Disease Stages

Figure 4 presents a simplified framework for longitudinal deep phenotyping across disease stages. In this review, deep phenotyping is distinguished from conventional multiparametric phenotyping by five minimum characteristics: (1) longitudinal assessment across prespecified disease stages; (2) an explicit hypothesis linking molecular lesions, mitochondrial or cellular responses, organ pathology, and whole-animal or patient-relevant function; (3) integration of repeated non-invasive measurements with stage-specific or terminal tissue endpoints; (4) alignment of sampling and interpretation with disease stage rather than chronological age alone; and (5) prespecification of primary outcomes with exploratory measurements clearly separated. A focused set of related endpoints can then test how a molecular lesion leads to functional impairment [5,8,10,72]. Exploratory omics can suggest mechanisms, but key findings require independent validation [17,72].

### 6.1. Molecular Lesions and Mitochondrial Function

Molecular phenotyping begins by identifying the function disrupted by the patient variant. Endpoints should be chosen according to the expected lesion and may include respiratory chain complex activity or assembly, substrate-dependent respiration, ATP production, membrane potential, redox state, mtDNA copy number, heteroplasmy, and mitochondrial translation [2,10,19,73]. Results from oxygen consumption or extracellular flux assays depend on cell or tissue type, substrate, permeabilization, coupling to ATP synthesis, and normalization method [73,74,75]. A single measure should not be treated as a proxy for overall mitochondrial function. Findings that depend on sample preparation should be checked using another preparation, rescue experiment, or orthogonal measurement. Omics should be used to identify candidate pathways rather than as proof of causation by itself [2,10,19,73].

### 6.2. Quantitative Pathology and Organ Function

Quantitative pathology identifies the affected cells and tissue structures; physiological measurements establish the consequences for organ function. Pathology should not rely only on representative images; lesion distribution, fibrosis, inflammation, cellular composition, and mitochondrial morphology should be quantified when relevant [2,8]. Organ function tests are needed to determine whether pathology translates into functional impairment. Endpoints may include cardiac function, respiration, liver function, muscle strength, exercise tolerance, neurophysiology, and visual or auditory responses, chosen according to the patient-relevant organ system [1,8,10,21,24,25,72,75]. When possible, organ function should be measured repeatedly in the same animals and linked to terminal pathology. Molecular or structural improvement should not be equated with functional recovery, especially in therapeutic studies [10].

### 6.3. Whole-Animal Function, Behavior, and Home-Cage Assessment

Whole-animal assessment examines how the disease affects behavior and general condition. In neuromuscular models, primary endpoints should be selected from motor coordination, strength, gait, endurance, sensation, and cognition according to the disease hypothesis [1,8,76]. Behavioral performance is shaped by motor capacity, sensory function, motivation, body temperature, circadian rhythm, and handling stress, so poor test performance should not be interpreted immediately as cognitive impairment [8,72,76,77]. Home-cage monitoring and markerless pose estimation can reduce experimenter intervention and quantify activity, feeding, sleep, gait, and posture over time [72,78,79,80]. High-dimensional behavioral features, however, can lead to overfitting and selective reporting; they should be separated from prespecified primary endpoints and replicated in independent cohorts [78,79,80].

### 6.4. Metabolism, Omics, and Disease-Stage Classification over Time

Metabolic studies can relate mitochondrial defects in individual tissues to changes in whole-body metabolism and disease progression. Cellular measures such as respiration, ATP production, membrane potential, and redox state can be related to whole-animal oxygen consumption, respiratory exchange ratio, energy expenditure, and activity at corresponding disease stages [1,10,19]. Respiration measurements are sensitive to substrates, ADP, uncouplers, inhibitors, temperature, sample preparation, and normalization, all of which should be reported [73,74,75]. Single-cell analysis and spatial transcriptomics can identify cell-type-specific responses and map the local spread of metabolic stress within heterogeneous tissues, but they are influenced by tissue dissociation, storage, nuclear isolation, sequencing depth, batch effects, and cell composition [81]. Measurements should follow the baseline, early change, established disease, and post-intervention stages, with primary and exploratory endpoints specified separately [17,72].

### 6.5. Setting Endpoints Based on Patient Information

Patient-relevant readouts are defined here as endpoints that correspond to clinical phenotypes, biomarkers, imaging findings, functional outcomes, or natural-history measures and can be measured in patients, animal models, or related model systems. Mapping patient endpoints onto animal-measurable endpoints helps interpret preclinical results. Correspondence can be organized at several levels: patient variants or protein abnormalities at the molecular level; respiratory chain function, ATP production, and membrane potential at the cellular level; pathology, fibrosis, and cell loss at the tissue level; cardiac function, muscle strength, exercise tolerance, and visual or auditory responses at the organ level; activity, behavior, body weight, and survival at the whole-animal level; and blood, imaging, or functional biomarkers at the clinical level. GDF-15 and FGF-21, for example, may become pharmacodynamic readouts in animal models if tissue source, stage dependence, and treatment responsiveness are established [82,83]. MRI, animal equivalents of the 6-min walk test (6MWT), cardiac function, neurological function, and natural-history indices from patient registries can also guide endpoint selection. Examples are summarized in Table 5.

Patient-derived iPSCs, differentiated cells, organoids, and organ-on-chip systems can replace animal experiments when the question concerns variant function, cell-autonomous mechanisms, selected rescue experiments, or screening in a human genetic background [84]. They can also complement whole-animal work by defining mechanisms and candidate endpoints. Animal models remain necessary when the claim requires integrated development, systemic metabolism, immunity, pharmacokinetics, long-term natural history, inter-organ interactions, or whole-animal safety. Recent pluripotent-stem-cell work in Leigh syndrome illustrates a staged approach in which patient-derived neural cells and brain organoids identify candidate therapies for subsequent evaluation in mammalian models and patient observations [85]. For gene-therapy studies, in vivo models are also important for evaluating delivery, biodistribution, and systemic safety [86]. The choice between replacement and complementation should therefore be stated for each research question rather than treated as a fixed property of a model system.

Animal studies can identify stages at which disease remains amenable to treatment, outcomes that change during a feasible treatment period, and biomarkers of target engagement or organ function. These findings can inform dose selection, assessment of target-organ exposure, and safety monitoring in early-phase trials. They may also help select functional outcomes for later trials. Figure 5 distinguishes the types of evidence provided by these measurements.

## 7. Lessons from Representative Models

The following examples illustrate how disease course, tissue specificity, and treatment mechanism influence experimental design. Table 6 summarizes the models and their main limitations.

Ndufs4-deficient mice provide a practical example of question-driven design in a canonical primary mitochondrial disease. Whole-body knockout models support a linked assessment of survival, respiration, motor decline, and neuropathology, whereas brain- or neuronal-population-specific deletion tests regional and circuit vulnerability [23,26,27,87,88,89]. Disease stage and treatment mechanism then determine the endpoints: rapamycin can be evaluated through pathway modulation and downstream functional outcomes, while chronic hypoxia improves survival, body weight, body temperature, behavior, neuropathology, and disease biomarkers without restoring the absent NDUFS4 protein or complex I activity [32,33,90]. Thus, a biologically effective intervention need not correct every upstream defect. Conversely, improvement in an upstream molecular endpoint alone cannot establish organ or whole-animal benefit. The severe early-lethal phenotype also limits extrapolation to milder or slowly progressive Leigh syndrome.

Polg mutator and Twinkle mutant mice demonstrate how disease timing shapes interpretation [62,64,91]. Polg mutator mice are useful for studying the accumulation of mtDNA mutations, tissue dysfunction, and aging biology, but they do not reproduce a specific patient allele [62,91]. Twinkle mutant mice show progressive mtDNA deletions and late-onset pathology, making them suitable for studying gradual transitions and intervention timing that are difficult to observe in rapidly fatal models [64].

Conditional models of Tfam, Cox10, and Lrpprc show that the same category of mitochondrial dysfunction can cause different pathology depending on organ environment, including heart, skeletal muscle, and liver [21,24,25,28,29,92,93]. These models are useful for studying organ vulnerability and the temporal order of pathology. However, many are based on complete gene deletion and may model more severe defects than the hypomorphic or missense variants common in patients.

Opa1 models and heteroplasmic mtDNA models show how endpoint selection affects conclusions [3,11,12,36,37,38,39,40,41,94,95]. In Opa1 Q285STOP heterozygous mice, dendritic changes in retinal ganglion cells precede obvious cell loss, indicating that neural connectivity can be an important sensory endpoint [94]. Heteroplasmic mtDNA models require the quantification of mutant load in relevant tissues and cell types; disease is determined by thresholds and tissue-specific mutant load rather than by variant presence alone [3,11,12,36,37,38,39,40,41].

Studies of Tk2 H126N knock-in mice and liver-targeted rescue in Mpv17-deficient mice illustrate the need to consider delivery and treatment timing alongside efficacy [34,35,96,97]. Molecular and pathological measurements should be assessed together with organ function, whole-animal outcomes, and safety at the exposure achieved.

Table 6 brings these examples together for a comparison of variant effects, organ vulnerability, disease course, and treatment response.

**Table 6:** Major mitochondrial diseases/genes and corresponding model systems. Abbreviations: KI, knock-in; KO, knockout.

Disease/Pathology/Gene	Main Model Systems	Assessable Pathology/Endpoints	Useful Purpose	Key Cautions
Leigh-like Complex I deficiency NDUFS4	Whole-body Ndufs4 KO, brain-specific and neuronal population-specific mouse models; deletion of NUYM, the Yarrowia lipolytica ortholog of human NDUFS4; patient iPSC systems [23,26,27,87,88,89,98].	Progressive encephalopathy, brain-region lesions, breathing dysfunction, motor decline, survival, treatment response.	Neural vulnerability, treatment timing, mTOR inhibition, hypoxia, metabolic interventions.	Skewed toward severe NDUFS4 disease; do not generalize to all Leigh syndrome or mild/long-term disease.
POLG-related disease POLG	Polg mutator, yeast, worm, zebrafish, patient cells.	mtDNA mutation accumulation, tissue dysfunction, premature aging-like phenotype, neuromuscular changes.	Mutation burden, mtDNA maintenance, modifier discovery, aging biology.	Not a single patient allele; do not treat as a complete model of POLG-related disease.
Progressive external ophthalmoplegia TWNK	Twinkle mutant mice and patient-derived or engineered cell systems [64].	Multiple mtDNA deletions, late-onset myopathy, motor function, muscle pathology.	Longitudinal natural history, intervention timing, muscle pathology, slowly progressive disease.	Slow progression requires long-term studies; design differs from early-lethal models.
mtDNA depletion syndrome TK2	Tk2 H126N KI and nucleoside/nucleotide supplementation models.	deoxyribonucleoside triphosphate (dNTP) imbalance, mtDNA depletion, muscle/neural phenotype, survival, pathology.	Preclinical evaluation of metabolic supplementation, route and timing.	Results depend on delivery route, exposure, and disease stage; separate molecular from functional improvement.
Hepatocerebral mtDNA depletion MPV17	Mpv17 KO and adeno-associated virus (AAV) liver replacement model.	Liver failure, mtDNA depletion, metabolic abnormalities, diet-induced liver injury.	Liver-targeted gene therapy, organ targeting, hepatic disease.	Neurological disease and long-term safety require separate assessment; liver improvement is not systemic benefit.
Mitochondrial myopathy COX10 and related genes	Skeletal muscle-specific Cox10 KO and tissue-specific Tfam models.	Exercise intolerance, COX deficiency, muscle pathology, strength, endurance.	Skeletal muscle respiratory-chain dysfunction, muscle function, tissue-specific requirement.	Complete deletion may be more severe than patient missense variants; Cre driver and fiber type matter.
Dominant optic atrophy OPA1	Opa1 mutant mice, retinal/optic nerve assessment.	Optic atrophy, retinal ganglion cell vulnerability, visual dysfunction, dendritic changes before cell death.	Sensory deep phenotyping, connectivity, longitudinal visual function.	Interpretation depends on allele type; retinal findings should not be directly equated with human symptoms.
Heteroplasmic mtDNA disease	Mito-mouse, transmitochondrial mouse, mtDNA editing models.	Threshold effects, maternal transmission, tissue segregation, cell-type-specific mutant load.	mtDNA-specific pathology, heteroplasmy control, mtDNA editing therapy.	Cell-type mutant load and precise editing are difficult; blood may not represent affected organs.
Leigh syndrome SURF1	Surf1-null mice and cloned SURF1-knockout pigs [20,57].	Cytochrome c oxidase deficiency, growth, neuromuscular phenotype, survival, and species-dependent severity.	Cross-species assessment of compensation and systemic disease severity.	The relatively mild mouse phenotype does not predict the severe lethal phenotype in pigs; cost and small cohorts constrain large-animal studies.

## 8. Multidomain Framework for Preclinical Therapeutic Evidence

Figure 5 presents complementary domains of preclinical therapeutic evidence without ranking them in a fixed sequence. Domain 1 is direct target engagement, demonstrated by evidence such as target binding, target occupancy, or proximal modulation of the intended molecular target. Target-organ exposure at a potentially active concentration is necessary for interpreting therapeutic effects but does not by itself demonstrate direct target engagement. Exposure should therefore be considered a cross-cutting requirement across all evidence domains. Domain 2 is molecular correction, including restoration of RNA or protein, correction of a pathogenic allele, or a change in edited mtDNA fraction or heteroplasmy. Domain 3 is a downstream pharmacodynamic or mitochondrial response, including pathway modulation, respiration, ATP production, membrane potential, complex activity, metabolite change, or cellular rescue. These measurements address distinct aspects of treatment action and should be interpreted separately [10].

Domain 4 is organ-level structural or functional improvement, such as cardiac performance, muscle strength, respiratory or neurological function, hearing, liver function, or histopathology linked to function. Domain 5 is whole-animal or patient-relevant benefit, including survival, behavior, exercise capacity, systemic function, or another outcome that corresponds directly to patient experience. Biomarkers are not assigned automatically to the highest domain: their interpretation depends on whether they demonstrate target engagement, biological activity, organ injury, or a validated relationship with function or clinical outcome. A stronger therapeutic claim is supported by a prespecified, mechanistically coherent combination of domains, but an intervention need not improve them in a fixed sequence.

Exposure, safety, and independent replication apply across all evidence domains. The hypoxia studies in Ndufs4-deficient mice are particularly informative because substantial functional and survival benefits occurred without restoration of complex I activity [33,90]. For mtDNA editing, edited fraction and molecular or metabolic rescue must be distinguished from organ-level efficacy and safety. In the AAV9-DdCBE study, compensatory editing improved molecular and metabolic measures, but the highest vector dose produced severe adverse effects associated with extensive mtDNA off-target editing [48]. The interpretation of treatment effects therefore depends on the mechanism and the outcomes measured, as summarized in Figure 5.

## 9. Preclinical Study Design, Reproducibility, and the 3Rs

Preclinical studies should define primary endpoints, disease stage, treatment duration, exclusion and stopping criteria, and statistical analysis before the experiments begin [17]. Each of the 3Rs has practical implications for this design. Replacement requires using non-animal systems whenever they can answer the prespecified question; Reduction requires an appropriate experimental unit, efficient longitudinal designs, and sample-size planning without pseudoreplication; and Refinement requires procedures and endpoints that minimize pain, distress, and non-specific physiological disruption. Randomization, blinding, sex, litter, cage, cohort, and measurement batch should be incorporated into allocation and analysis [14,15,17,99]. Negative studies should be interpreted only after verifying target-organ exposure, target engagement, assay sensitivity, and disease-stage appropriateness [10].

Replication should be planned from the outset [14,15,16]. An independent cohort, a separate experimental batch, another facility, and a complementary allele each test a different source of variation. Restricting sex, age, genetic background, and husbandry too tightly can produce conclusions that hold only under narrow conditions. Where the study objective permits, controlled variation in these factors can improve generalizability [14,15,16].

When properly designed, deep phenotyping can support both reproducibility and the 3Rs [17,72]. Longitudinal imaging, home-cage monitoring, microsampling, and repeated physiological measures can obtain time-resolved information from the same individuals and reduce animal use while quantifying individual variation and disease progression. Temperature management, non-aversive handling, specified anesthesia, recovery monitoring, and prespecified endpoints stabilize animal condition and reduce stress-, hypothermia-, and procedure-related variation [17,71,76]. Table 7 summarizes the minimum information that should be reported for preclinical mitochondrial disease animal studies.

## 10. Future Perspectives and Unresolved Issues

New editing methods allow for more precise construction of hypomorphic, conditional, humanized, and mtDNA models [9,42,43,44,45,46,47,48,49,50,56,100], but greater editing activity does not by itself establish a useful or safe model. TALEDs can cause extensive transcriptome-wide RNA off-target editing. Engineered deaminase variants reduce these effects, although RNA, mtDNA, nuclear-DNA, and bystander edits still require assessment [101]. Delivery and dose also matter: AAV9-DdCBE-mediated compensatory editing improved molecular and metabolic outcomes in mice, but the highest vector dose caused severe adverse effects associated with extensive mtDNA off-target editing [48]. Editing efficiency, functional outcomes, and adverse effects should therefore be reported separately.

Human iPSCs, differentiated cells, organoids, and organ-on-chip systems can replace animal experiments for suitable questions about variant function, cell-specific mechanisms, drug response, and rescue [84]. Work in Leigh syndrome illustrates how screening in patient-derived neural cells and brain organoids can identify candidates for further evaluation in mammalian models and patient observations [85]. Animal studies remain relevant when the question requires integrated physiology, inter-organ interactions, pharmacokinetics, behavior, or long-term systemic safety. The experimental requirements, rather than a fixed order of model systems, should determine whether an animal study is needed.

For such studies, a useful next step is to identify which cellular findings predict organ dysfunction or treatment response in vivo and which depend on the culture conditions.

AI- and machine-learning-based image analysis, home-cage monitoring, markerless pose estimation, and markerless behavioral analysis may reduce experimenter-dependent variability and detect subtle functional decline over time [72,78,79,80]. Methods such as DeepLabCut track body parts from ordinary video without reflective markers or sensors and can quantify gait, posture, and activity [78,79]. High-dimensional feature sets, however, carry risks of overfitting and selective reporting. Primary endpoints should be prespecified, findings should be replicated in independent cohorts, and results should be aggregated into interpretable functional domains [78,79,80].

Comparisons between laboratories will require consistent reporting of genetic background, husbandry, heteroplasmy, and phenotyping methods, together with accessible protocols and data [5,17,18]. Agreement on a core set of informative functional measurements would make these comparisons more useful without adding unnecessary tests.

## 11. Conclusions

Mitochondrial disease studies are most informative when the genetic design, disease course, and endpoints fit the question under investigation. Yeast and human cell systems can resolve many molecular and cell-autonomous questions. Animal models are needed where the experiment depends on integrated physiology, disease progression, delivery, or systemic safety. Therapeutic evaluation should distinguish molecular effects from functional benefit and assess both in relation to exposure and adverse effects.

Differences between species and alleles, as illustrated by the IARS1 studies, can help explain why a model develops a particular phenotype or remains relatively unaffected. Longitudinal phenotyping is useful when it tests a defined relationship between the molecular defect and subsequent functional decline. Prespecified outcomes, alignment with patient natural history, and independent replication make the resulting evidence easier to interpret and more useful for preclinical decisions.

## Figures and Tables

**Figure 1:**
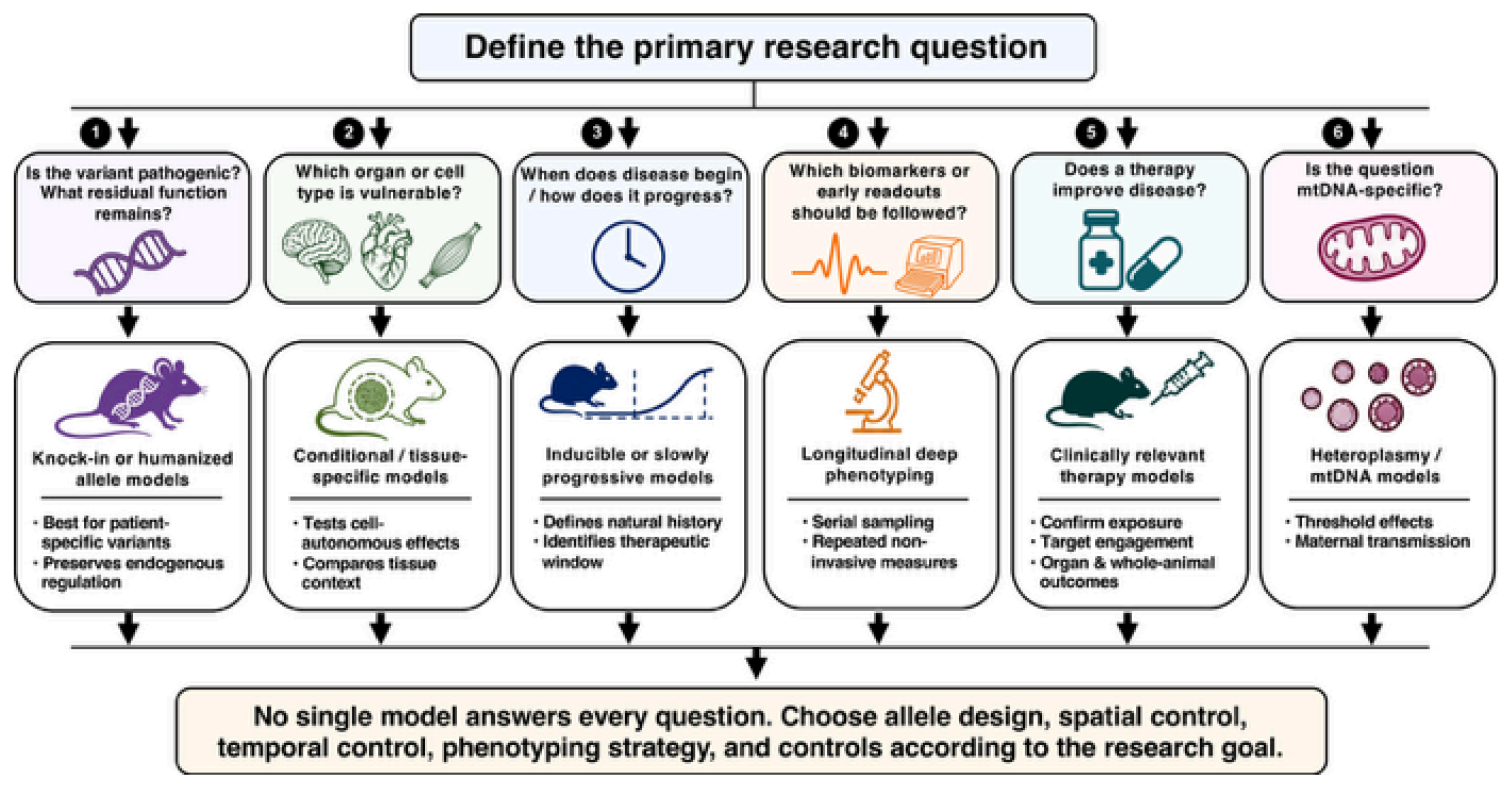
Research question-driven model selection in mitochondrial disease research. Six research aims are linked to suitable model types and design choices: assessing variant pathogenicity or residual function, identifying vulnerable organs or cells, defining disease onset and progression, selecting biomarkers or early readouts, testing therapeutic efficacy, and investigating mtDNA-specific mechanisms. Figure 2 shows how additional model systems can contribute to these studies. mtDNA, mitochondrial DNA.

**Figure 2:**
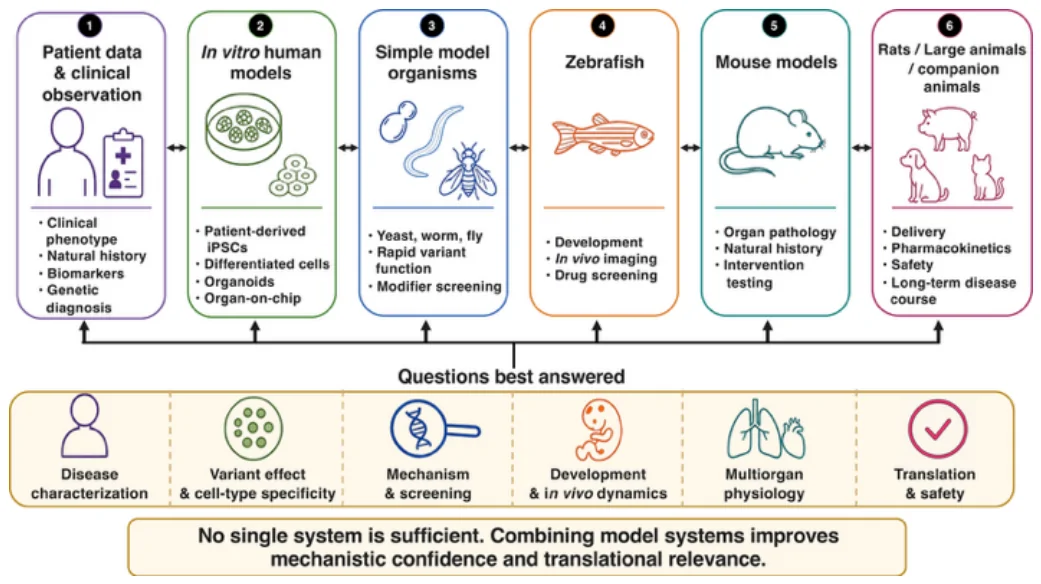
Complementary model systems for mitochondrial disease research. Yeast and cultured cells permit the rapid testing of variant function and rescue. Worms, flies, and zebrafish are useful for studies of development, movement, neural function, genetic modifiers, and drug screening. Mice allow the assessment of organ pathology, disease progression, and treatment timing. Rats, large animals, and naturally affected companion animals provide additional opportunities to study pharmacokinetics, delivery, safety, and long-term disease course. Findings in patients, human cell systems, and animals can inform further experiments in any of these systems. iPSC, induced pluripotent stem cell.

**Figure 3:**
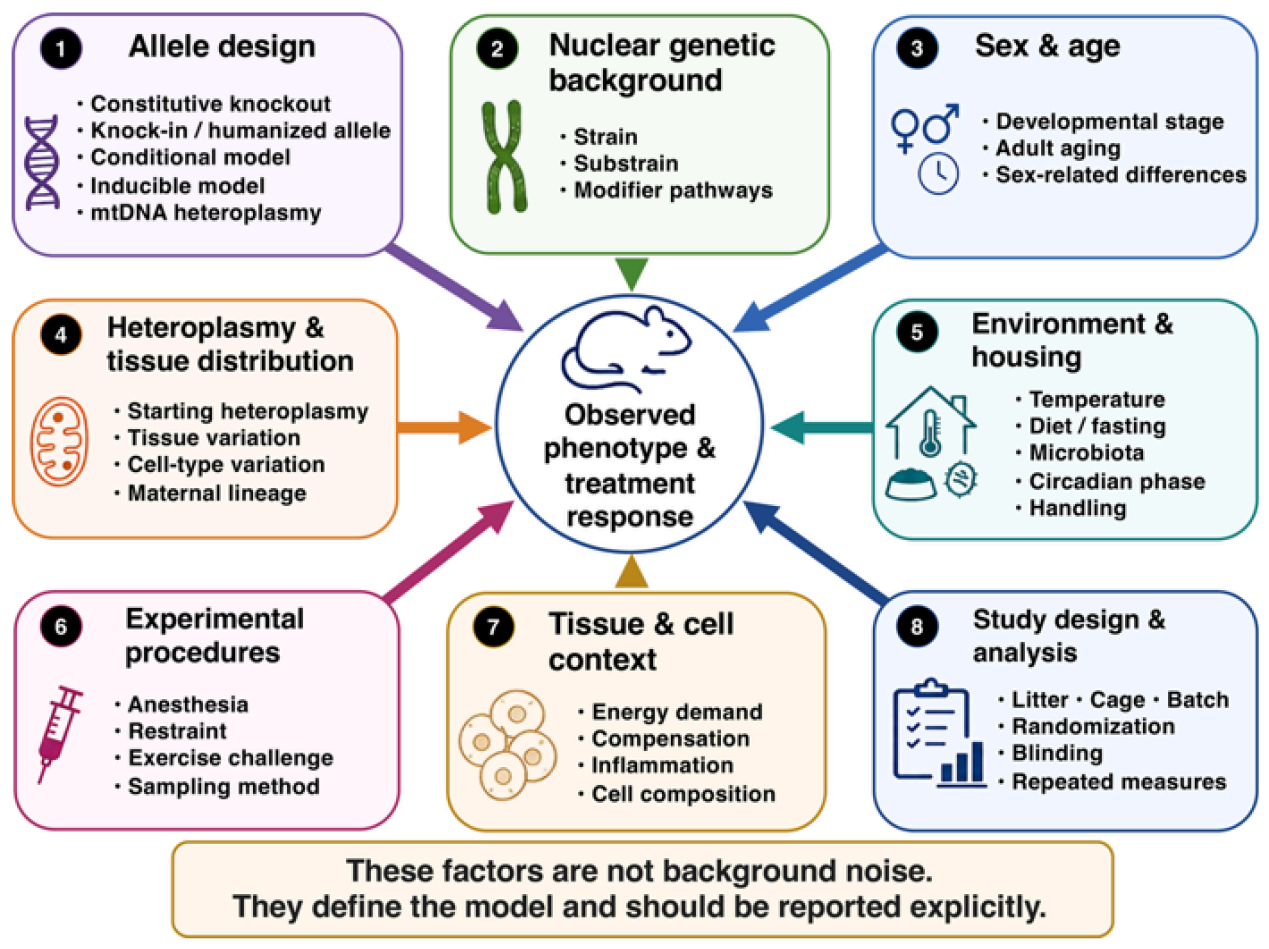
Factors that modify phenotype and therapeutic response in mitochondrial disease models. The introduced allele is central, but observed outcomes are also influenced by genetic background, sex, age, heteroplasmy, maternal lineage, tissue and cellular environment, husbandry, microbiota, handling, measurement procedures such as anesthesia, restraint, and sampling, and study design including randomization, blinding, and batch structure. These factors should be recorded, controlled, or incorporated into the analysis as components of the model.

**Figure 4:**
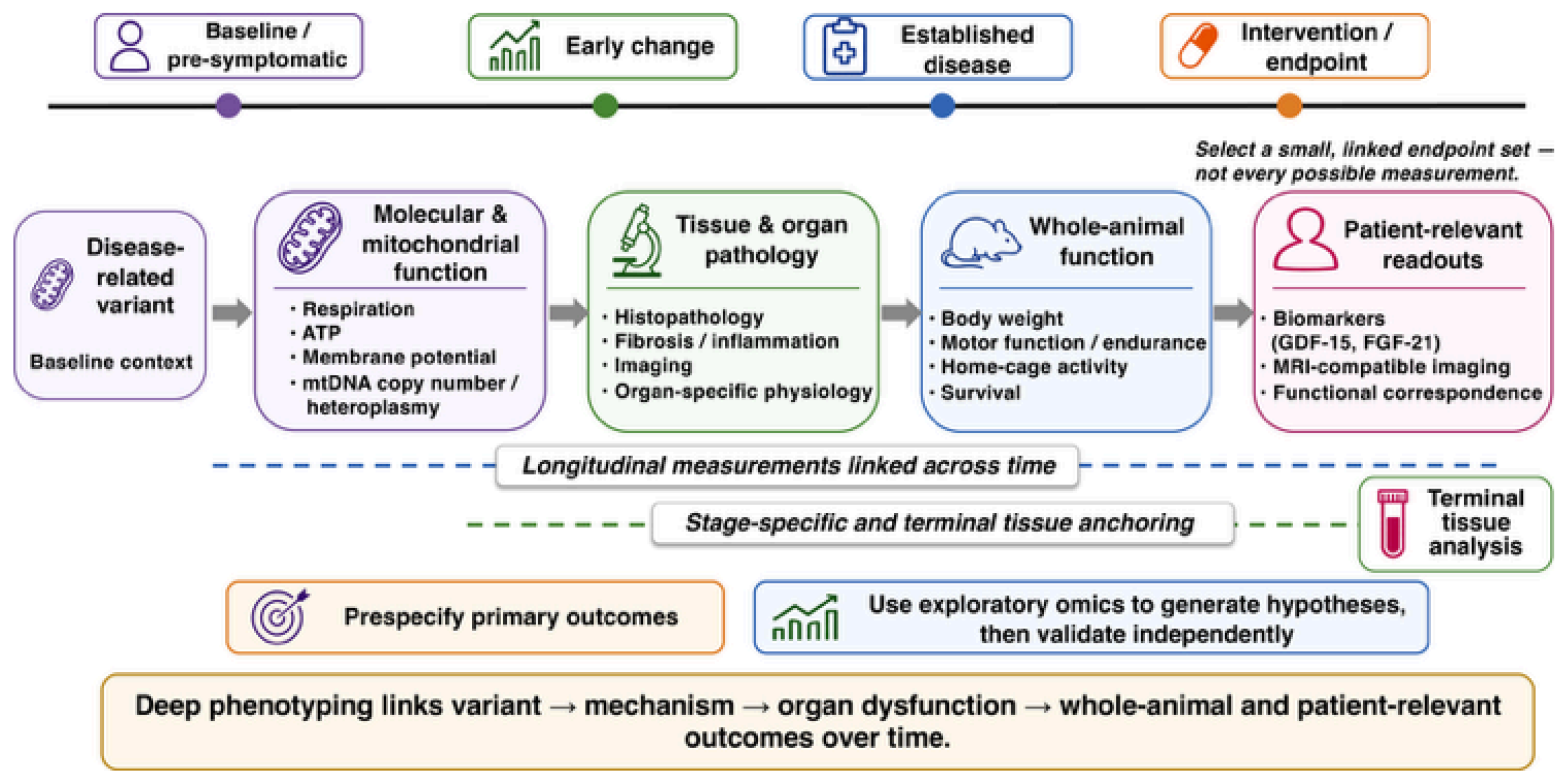
Longitudinal deep phenotyping across disease stages. Deep phenotyping is defined here by longitudinal assessment, prespecified links among measurement levels, integration of repeated and terminal endpoints, disease-stage alignment, and separation of primary from exploratory outcomes. The framework links a patient variant and baseline context to molecular and mitochondrial function, tissue and organ pathology, whole-animal function, and patient-relevant readouts from the presymptomatic stage through established disease and intervention. ATP, adenosine triphosphate; GDF-15, growth differentiation factor 15; FGF-21, fibroblast growth factor 21; MRI, magnetic resonance imaging; mtDNA, mitochondrial DNA.

**Figure 5:**
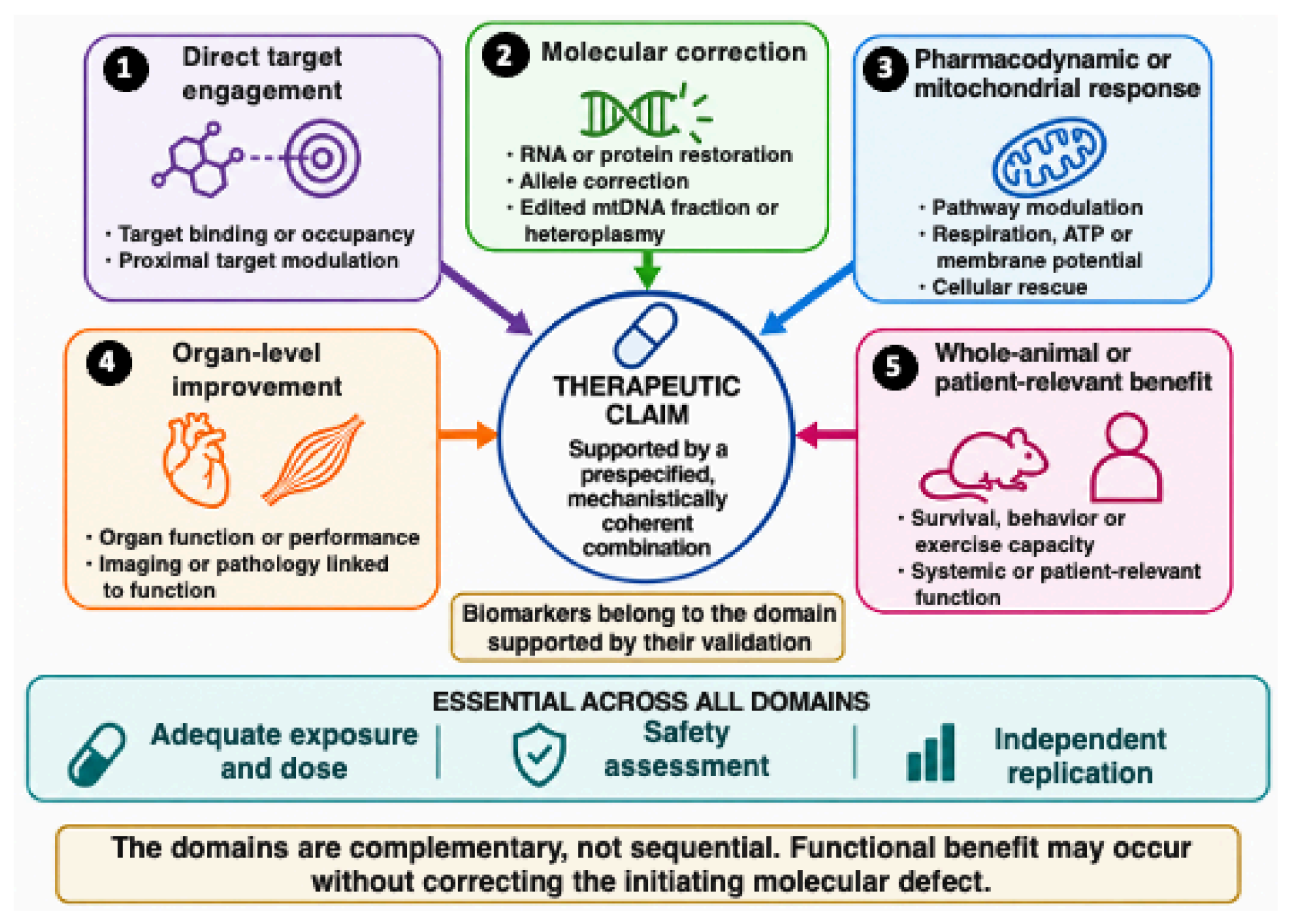
Multidomain framework for preclinical therapeutic evidence. Five evidence domains are distinguished: direct target engagement; molecular correction; downstream pharmacodynamic, mitochondrial, or cellular response; organ-level structural or functional improvement; and whole-animal or patient-relevant benefit. The domains are complementary rather than a mandatory sequence. A treatment may provide functional benefit without correcting the initiating molecular defect, as illustrated by hypoxia in Ndufs4-deficient mice. Biomarkers should be assigned according to what they demonstrate rather than automatically being treated as high-level evidence. Exposure, dose, safety, and independent replication apply across all domains. ATP, adenosine triphosphate; mtDNA, mitochondrial DNA.

**Table 1:** Major genetic designs used in mitochondrial disease animal models. The table compares the questions each design can address, its strengths, limitations, and common applications.

Model Design	Questions Addressed	Main Strengths	Key Limitations	Useful Applications
**Constitutive knockout**	Is the gene required for development or survival? What happens after severe loss of function?	Whole-body gene loss can demonstrate essential gene function. Embryonic lethality or severe phenotype indicates importance for development or organ maintenance.	Embryonic lethality and developmental compensation are common. Complete loss may differ from patient missense or partial loss-of-function variants. Postnatal natural history and therapeutic windows may be difficult to study.	Gene-function discovery, assessment of essentiality, severe loss-of-function biology.
**Knock-in humanized allele**	Is a patient variant disease-related? Can therapies that depend on human sequences be evaluated?	Patient variants or human sequences can be studied near endogenous expression levels and patterns. Useful for residual function, progression, and allele-specific therapy.	Phenotypes may be mild, delayed, or influenced by species and background. Humanized alleles can be complex to generate and may attenuate phenotype.	Variant pathogenicity, precision therapy, allele-specific therapy, gene replacement, and nucleic acid therapy.
**Conditional tissue-specific**	Which organ or cell type is critical? Is the lesion cell-autonomous?	Genetic manipulation in selected organs or cells facilitates the analysis of organ-specific roles and avoids embryonic lethality.	Cre expression range, recombination efficiency, mosaicism, and Cre toxicity require controls. Tissue-restricted models should not be treated as full multisystem models.	Organ-specific pathology, cell-autonomous mechanisms, cellular and tissue vulnerability.
**Inducible**	When does perturbation drive disease? When should therapy begin?	Timing of gene perturbation can be controlled, allowing the separation of developmental from postnatal or adult pathology and testing of therapeutic windows.	Inducers, recombination variability, dose, and timing can confound interpretation. Inducer-only and Cre-only controls are important.	Disease time course, post-onset degeneration, timing of intervention.
**Heteroplasmic mtDNA**	What mutant mtDNA load causes disease? How do tissue differences and maternal transmission shape phenotype?	Captures coexistence of normal and mutant mtDNA and enables the study of thresholds, tissue differences, maternal inheritance, and heteroplasmy control.	Mutant load varies by tissue, cell type, and age. Blood or other easily sampled tissues may not represent affected organs.	mtDNA disease mechanisms, heteroplasmy control, mtDNA editing, tissue-specific threshold effects.

**Table 2:** Complementary model systems used in mitochondrial genetic disease research. The table compares biological scope, physiological relevance, and typical roles in staged validation. The final column provides qualitative descriptors of relative speed and resource requirement.

Model System	Main Advantages	Main Limitations	Useful Applications	Relative Speed and Resource Requirement
**Yeast**	Uses conserved mitochondrial pathways to test the effects of variants on respiration, mtDNA maintenance, and mitochondrial translation rapidly and at low cost [60,61,62].	Not an animal; cannot assess organs, behavior, development, pharmacokinetics, or systemic pathology. A negative result alone should not exclude pathogenicity.	Initial variant-function testing, complementation assays, respiration, mtDNA maintenance, candidate drug, or modifier screening.	Low cost; rapid.
***C. elegans***	Short generation time, transparency, strong genetics, lifespan, movement, and membrane-potential assays; suited to screening [2,51,52].	Cannot directly assess mammalian cardiopulmonary, renal, or hepatic function. Correspondence to patient multisystem disease is limited.	Stress responses, lifespan, movement, neurodegeneration, modifier genes, drug screening.	Low cost; rapid.
**Drosophila**	Tissue-specific genetics facilitate neural and muscle phenotypes, mitochondrial dynamics, and genetic interactions [2,53].	Anatomy, metabolism, and organ structure differ markedly from mammals; not suited to mammalian pharmacokinetics or organ function.	Neurodegeneration, muscle function, mitochondrial dynamics, genetic interactions, pathway discovery.	Low to medium cost; rapid.
**Zebrafish**	Transparent embryos, rapid development, in vivo imaging, CRISPR, and drug screening enable developmental, neural, and motor phenotypes [2,54,60].	Ectothermy, gene duplication, gill respiration, and exposure routes differ from mammals; mammalian exposure and organ function cannot be assumed.	Developmental defects, neural pathology, motor phenotype, live imaging, drug candidate screening.	Medium cost; medium speed.
**Rat**	Larger size supports repeated sampling, surgery, physiological monitoring, pharmacokinetics, and complex behavior; an engineered heteroplasmic mtDNA rat developed hearing impairment [55,56].	Fewer mitochondrial disease lines and shared resources than mice; higher costs for generation and maintenance.	Cardiopulmonary physiology, neurobehavior, long-term monitoring, dose setting, pharmacokinetics.	Medium cost; medium speed.
**Cattle/naturally affected livestock**	Naturally occurring variants and perinatal disease can be studied in a comparative medicine context [63].	Long generation time, high cost, small sample sizes, and limited control of genetic and environmental background.	Natural history, perinatal and growth disorders, species comparisons, human-large animal-mouse comparisons.	Medium to high cost; long-term.
**Pig**	Human-like organ size and physiology support imaging, delivery, and safety studies; SURF1-knockout pigs developed severe systemic disease unlike the relatively mild Surf1-null mouse phenotype [20,57,58].	High production and husbandry costs, long generation time, facility requirements, and few available disease models.	Gene and nucleic-acid therapy delivery, organ targeting, pharmacokinetics, safety, procedural development.	High cost; long-term.
**Companion animals**	Naturally occurring disease can be observed under clinical management with comorbidities and long-term treatment responses [59].	Heterogeneous cases, environmental differences, small sample sizes, consent and ethics constraints; less experimental control than laboratory models.	Comparative clinical research, biomarkers, treatment response in clinical settings, safety support.	Clinically relevant but case-dependent.

**Table 3:** Model selection according to the main experimental objective. When feasible, rescue refers to restoration using the wild-type allele or an independently validated corrective intervention.

Research Purpose	Suitable Models	What to Confirm	Pitfalls	Controls
**Test whether a patient variant is disease-related**	Knock-in or humanized allele carrying the patient variant; yeast or cell-based functional testing when useful.	Expression of the mutant allele, protein amount, localization, biochemical effect, complementation or independent validation.	Do not treat a complete knockout phenotype as proof that a patient missense variant is pathogenic.	Wild-type, littermate, ideally isogenic controls; rescue with the wild-type allele or an independently validated corrective intervention when feasible.
**Identify the critical organ or cell type**	Conditional/tissue-specific model with a clearly defined Cre driver.	Recombination in target tissue, lack of unintended non-target effects, systemic status.	Do not treat a tissue-restricted model as a full multisystem model; consider Cre effects.	Cre-negative littermates, Cre-positive controls, non-target organ analysis, recombination efficiency.
**Define when and in what order disease progresses**	Viable progressive model; inducible models or slow-progressing knock-in models are useful.	Pre-symptomatic, early-change, established-disease, and endpoint stages on one timeline.	Do not interpret only by age; align by body weight, functional decline, or pathology.	Littermate controls, repeated measures within individuals, disease-stage-matched controls.
**Discover biomarkers**	Model with stable disease course and repeat sampling; matched tissue and blood/urine readouts.	Association with disease stage, organ pathology, functional decline, treatment response, and sampling effects.	Do not define usefulness from a single time-point difference; confirm tissue source and stage dependence.	Stage-matched controls, repeat-sampling controls, independent cohort when possible.
**Test therapeutic efficacy**	Model reproducing relevant variant, delivery route, and treatment timing; target pathway must be impaired.	Target-organ exposure, target engagement, mitochondrial function, organ function, and whole-animal outcome.	Do not equate molecular correction with functional recovery; consider intervention too late in disease.	Vehicle, untreated, exposure-confirmation, and disease-stage-matched controls.
**Assess safety**	Model with appropriate age, sex, background, and ability to evaluate off-target organs; long-term observation when possible.	Clinical chemistry, pathology, behavior, developmental and long-term effects, off-target organs, immune response, off-target editing.	Do not assess safety only in the diseased organ; absence of short-term toxicity is not long-term safety.	Age-, sex-, background-matched controls, off-target organ analysis, dosing-only controls.

**Table 4:** Comparative medical evidence across species, systems, and alleles for IARS1.

Species/System/Allele	Main Findings	What this System Shows	Representative Reference	Role
Japanese Black cattle naturally occurring homozygous IARS1 p.Val79Leu	Perinatal low body weight, weakness, reduced muscle strength, and difficulty nursing.	Effect of a natural allele on perinatal growth and whole-animal condition.	Hirano et al. [63]	Individual/natural history.
Yeast complementation assays for human IARS1 variants	Rapid testing of variant-related functional reduction.	Whether the variant itself reduces IARS1 function more quickly than animal experiments.	Kopajtich et al. [69]	Molecular function.
Mouse Iars1 V79L, null, and Y148C/R444 * alleles	The hypomorphic allele causes growth and liver phenotypes; complete deletion is embryonic lethal; the compound-heterozygous allele causes pulmonary surfactant accumulation.	Allele severity and cellular environment determine which questions and organs can be studied.	Watanabe et al. [66,67]; Zhou et al. [68]	Allelic series, development, and organ context.
Human biallelic IARS1 variants	Prenatal growth retardation, intellectual disability, hypotonia, infantile hepatopathy.	Benchmark for whether animal, yeast, and cell data correspond to patient phenotype.	Kopajtich et al. [69]	Patient correspondence.

**Note**: Because IARS1 encodes a cytosolic isoleucyl-tRNA synthetase, the primary defect is expected to be reduced cytosolic protein synthesis. The models complement one another by showing how this defect appears as growth failure, organ pathology, cellular dysfunction, or patient phenotype. R444*: ‘*’ denotes a stop codon.

**Table 5:** Examples of patient-to-animal endpoint translation. Aligning patient blood, imaging, functional, and natural-history measures with animal-model endpoints increases the patient relevance of preclinical results.

Patient Evaluation Domain	Representative Patient Endpoints	Corresponding Animal Endpoints	Interpretation
**Blood/metabolic biomarkers**	GDF-15, FGF-21, lactate, clinical chemistry.	Serum/plasma markers, urine markers, organ-specific expression, post-treatment change.	Assess disease stage, tissue source, and relationship to functional impairment, not only group differences.
**Imaging/tissue injury**	MRI, organ imaging, muscle, brain, and cardiac findings.	Small-animal MRI, echocardiography, histopathology, fibrosis, cell loss.	Interpret imaging and pathology together with organ and whole-animal function.
**Motor/muscle function**	6MWT, muscle strength, exercise tolerance, fatigue.	Grip strength, treadmill, rotarod, activity, respiratory exchange ratio.	Exclude effects of motivation, body size, sensation, and handling.
**Cardiac/respiratory/neurological function**	Cardiac function, respiration, neurological signs, vision, hearing.	Echocardiography, respiratory monitoring, neurobehavior, visual and auditory responses.	Do not equate improvement in one organ with systemic benefit; match the patient-affected system.
**Natural history/systemic status**	Age at onset, disease stage, body weight, hospitalization, registry data.	Baseline, early-change, established-disease, and post-intervention longitudinal measures.	Compare animals by disease stage as well as chronological age.

**Table 7:** Minimum reporting items for rigorous mitochondrial disease animal studies. This disease-specific checklist complements rather than replaces ARRIVE 2.0 [17]. The checkbox (☐) indicates an item to be confirmed before submission.

Domain	Minimum Information	Why it Matters	Pre-Submission Check
**Genetic model**	Allele name, editing details, founder line, zygosity, expression/protein confirmation, off-target editing strategy.	Clarifies what was manipulated and aligns interpretation.	**☐**
**Genetic background**	Strain/substrain, backcrossing, breeding scheme, littermate controls.	Background genes can modify phenotype and treatment response.	**☐**
**Animals**	Sex, age, developmental stage, body weight, health status, exclusion criteria.	Avoids confusing sex, age, or health status with disease or treatment effects.	**☐**
**mtDNA**	Maternal lineage, heteroplasmy assay, tissues measured, sampling time, cell-type analysis.	Mutant mtDNA load changes by tissue, age, and cell type and directly shapes interpretation.	**☐**
**Environment**	Temperature, diet, fasting, light/dark cycle, cage density, enrichment, microbiota information.	Affects energy demand, activity, stress response, and mitochondrial phenotype.	**☐**
**Procedures**	Handling, anesthesia, warming, blood/tissue sampling, test order, recovery conditions.	Procedures can alter respiration, body temperature, metabolism, and behavior.	**☐**
**Study design**	Primary endpoints, experimental unit, randomization, blinding, sample-size rationale, exclusion and stopping rules.	Reduces bias, selective reporting, and overestimation; makes negative results interpretable.	**☐**
**Analysis**	Repeated-measures model, litter/cage/batch structure, prevention of pseudoreplication, prespecified handling of missing longitudinal data, and separation of confirmatory from exploratory analyses.	Reflects clustering and time course, prevents inflated sample size, and avoids bias from informative dropout or selective analysis.	**☐**
**Animal condition and 3Rs**	Health monitoring, body temperature and recovery, non-aversive handling, predefined endpoints, adverse events, 3R considerations.	Reduces non-specific effects such as stress, hypothermia, procedure-related variation, and severe deterioration.	**☐**
Replication	Numbers of independent biological replicates and technical replicates, with repeated measurements distinguished from independent observations.	Clarifies the strength of replication and prevents technical measurements from being treated as additional experimental units.	☐
Protocols, data, and code	Persistent access to detailed protocols, raw and processed data, metadata, and analysis code, with justified restrictions where necessary.	Supports reproducibility, reanalysis, and transparent interpretation.	☐

## Data Availability

No new data were generated or analyzed in this review. Data sharing is therefore not applicable.
